# Fn14, a Downstream Target of the TGF-β Signaling Pathway, Regulates Fibroblast Activation

**DOI:** 10.1371/journal.pone.0143802

**Published:** 2015-12-01

**Authors:** Shaoxian Chen, Juli Liu, Min Yang, Wen Lai, Litong Ye, Jing Chen, Xinghua Hou, Hong Ding, Wenwei Zhang, Yueheng Wu, Xiaoying Liu, Shufang Huang, Xiyong Yu, Dingzhang Xiao

**Affiliations:** 1 Medical Research Department of Guangdong General Hospital, Guangdong Academy of Medical Sciences, Guangzhou, Guangdong, China; 2 Key Laboratory of Regenerative Biology, South China Institute for Stem Cell Biology and Regenerative Medicine, Guangzhou Institutes of Biomedicine and Health, Chinese Academy of Sciences, Guangzhou, Guangdong, China; 3 Pharmacy Department of Guangdong General Hospital, Guangdong Academy of Medical Sciences, Guangzhou, Guangdong, China; 4 Burn and Wound Repair Surgery of Guangdong General Hospital, Guangdong Academy of Medical Sciences, Guangzhou, Guangdong, China; 5 Pharmacy Department of General Hospital of Guangzhou Military Command of PLA, Guangzhou, Guangdong, China; University of Louisville School of Medicine, UNITED STATES

## Abstract

Fibrosis, the hallmark of human injuries and diseases such as serious burns, is characterized by excessive collagen synthesis and myofibroblast accumulation. Transforming growth factor-β (TGF-β), a potent inducer of collagen synthesis, has been implicated in fibrosis in animals. In addition to TGF-β, fibroblast growth factor-inducible molecule 14 (Fn14) has been reported to play an important role in fibrotic diseases, such as cardiac fibrosis. However, the function and detailed regulatory mechanism of Fn14 in fibrosis are unclear. Here, we investigated the effect of Fn14 on the activation of human dermal fibroblasts. In normal dermal fibroblasts, TGF-β signaling increased collagen production and Fn14 expression. Furthermore, Fn14 siRNA blocked extracellular matrix gene expression; even when TGF-β signaling was activated by TGF-β1, fibroblast activation remained blocked in the presence of Fn14 siRNA. Overexpressing Fn14 increased extracellular matrix gene expression. In determining the molecular regulatory mechanism, we discovered that SMAD4, an important TGF-β signaling co-mediator, bound to the Fn14 promoter and activated Fn14 transcription. Taken together, these results indicate that the TGF-β signaling pathway activates Fn14 expression through the transcription factor SMAD4 and that activated Fn14 expression increases extracellular matrix synthesis and fibroblast activation. Therefore, Fn14 may represent a promising approach to preventing the excessive accumulation of collagen or ECM in skin fibrosis.

## Introduction

Fibrosis is characterized by the overproduction of collagen and other extracellular matrix (ECM) components and their accumulation in skin, lungs, and other tissues [1]. Excessive accumulation of collagen results in altered tissue architecture in injuries and disorders such as burns, systemic lupus erythematosus (SLE), scleroderma, keloids, hypertrophic scars, liver cirrhosis, and glomerulosclerosis [2–6]. Collagen consists mainly of type I and III collagen, which constitute approximately 95% of all known collagen types [7]. Fibroblasts produce ECM proteins as part of their fundamental role in normal wound repair [8]. Although fibrosis accounts for substantial morbidity and mortality in patients with injuries and diseases such as serious burns, its pathogenesis is not well understood, and there are currently no effective treatments. Many extracellular signals have been implicated in triggering and/or sustaining the process of fibrosis [9, 10]. Among them, the cytokine transforming growth factor-β (TGF-β) is the most potent inducer of fibroblast activation and ECM synthesis. Indeed, TGF-β is involved in physiological tissue repair and immune regulation. However, aberrant TGF-β signaling is associated with fibrosis and other pathological conditions [11].

Human Fn14, identified as the tumor necrosis factor-like weak inducer of apoptosis (TWEAK) receptor, is located at chromosome 16p13.3 [12]. Recent evidence indicated that fibroblast growth factor-inducible molecule 14 (Fn14) plays an important role in cardiac fibrosis [13–16] kidney fibrosis [17–19] and muscle fibrosis [20–23]. However, whether Fn14 plays a role in human skin fibrosis is still unknown.

In this study, we investigated the effect of Fn14 on ECM expression and fibroblast activation in human dermal fibroblasts (HDFs) and examined whether there is an interaction between Fn14 and TGF-β signaling. Our results provide evidence for a causative role of Fn14 in promoting ECM expression and HDF activation and demonstrate that the TGF-β signaling pathway promotes ECM synthesis by upregulating Fn14 expression.

## Materials and Methods

### Reagents

The TGF-β signaling pathway inhibitor SB431542 was purchased from Calbiochem (USA). Recombinant human TGF-β1 was obtained from PeproTech (USA). Cell culture reagents were purchased from Gibco (USA). Enhanced chemiluminescence reagents were obtained from Bio-Rad (USA). Protein extraction assay reagent was obtained from Roche (Swiss). TRIzol Reagent was purchased from Life Technologies (USA). Details on all the antibodies used in this study are included in S1 Text.

### Cell culture, transfection and tissues

Human fibroblasts were obtained from skin biopsies of healthy donor foreskin with informed consent and in compliance with the Institutional Review Board for Human Studies. In the experiments, dermal fibroblasts were obtained from five donors. Dermal fibroblasts were cultured from the biopsy specimens as described previously [24]. Briefly, cells were dissociated using 0.25% collagenase type I (Sigma, USA) and 0.05% DNase (Sigma, USA) in Dulbecco’s modified Eagle’s medium (DMEM) (Life Technologies, USA) with 20% fetal bovine serum (Life Technologies, USA). All of the cells were cultured in DMEM with 10% fetal bovine serum for all experiments. On day 2 after transfection with the p-Fn14 vector or Fn14 siRNA and treatment with or without TGF-β1 (10 ng/mL) or SB431542 (10 μmol/L), the cells were processed for real-time RT-PCR analysis or western blotting analysis. The inhibitor SB431542 was added to the cells before the addition of TGFβ1. The cells were treated for 48 h with TGF-β1.

All of the blood and skin tissues were collected from burn patients after 6 months of wound healing in Guangdong General Hospital. Adult burn patients between 20 and 50 years of age with deep burns (deep second (2b) or third degree burns) were selected. The study was conducted according to the principles of the Declaration of Helsinki and was approved by the Research Ethical Committee of the Guangdong General Hospital.

### Ethics statement

Written informed consent was obtained from all subjects who participated in this study. The study was conducted according to the principles of the Declaration of Helsinki and was approved by the Research Ethical Committee of the Guangdong General Hospital.

### Plasmid constructs

Human Fn14 cDNA was cloned into the pcDNA3.1 vector. pcDNA3.1 empty vector was used as a negative control. All transfections were performed using Sinofection reagent (Sino Biological Inc., China) according to the manufacturer’s instructions. The primer sequences for cloning the human Fn14 overexpressing-vector are provided in S1 Text.

### siRNA information

siRNA targeting human Fn14 (Fn14 siRNA) [25] was ordered from RiboBio (China). Negative control siRNA (GL2 siRNA) was also purchased from RiboBio (China), and Trans-EZ siRNA (Sunbio, China) was used to transfect dermal fibroblasts according to the manufacturer’s recommendations. The transfected siRNA concentration was 50 nM. The siRNA sequences used in this study are provided in the S1 Text.

### Immunoblotting analysis

Dermal fibroblasts were grown to 60% confluence and then subjected to different treatments or transfection. After the appropriate time period, the medium was removed, and the cells were processed. Protein expression was analyzed by western blotting with specific primary antibodies and horseradish peroxidase-conjugated secondary antibodies. Protein levels were quantitated by scanning densitometry using FluorChem 8900 software. Information on all the antibodies used in this study are provided in S1 Text.

### Total cellular RNA extraction, cDNA preparation, and quantitative real-time RT–PCR analysis

Total RNA was extracted using TRIzol Reagent. RNA quality was assessed by the agarose gel method, and approximately 1 μg of RNA was used to prepare cDNA using a First-Strand Synthesis Kit (Takara, Japan). Real-time RT–PCR was performed in triplicate using SYBR Green I (Takara, Japan) on an ABI 7500 machine (ABI, USA) using 1 μl of cDNA with β-actin as the internal control. The sequences of all the primers used in this study are provided in S1 Text. The relative gene expression was normalized to the expression of β-actin as an internal standard.

### Chromatin immunoprecipitation (ChIP)

ChIP assays were performed as previously described [26]. Briefly, cultured cells were cross-linked with 1% formaldehyde and incubated at 25°C for 15 min. Glycine (final concentration, 125 mM) was used to terminate the cross-linking. Washed cells were scraped and treated as described previously [26]. DNA was extracted and analyzed. A rabbit anti-SMAD4 antibody (Cell Signaling Technology, USA) and rabbit IgG (control; Cell Signaling Technology, USA) were used in these experiments. The primer sequences are provided in S1 Text.

### Statistical analysis

At least three independent experiments are shown, and data are presented as the mean ± SD (standard deviation). Independent experiments were conducted using donor cells from multiple donors. Statistical analyses were performed using SPSS 17.0 software. Statistical significance was determined using the 2-tailed Student’s *t* test when comparing 2 groups and ANOVA followed by *post-hoc* analysis with LSD or Dunnett’s T3 test when comparing >2 groups. A p value <0.05 was considered statistically significant.

## Results

### Fn14 expression is regulated by TGF-β signaling

**Fig 1:**
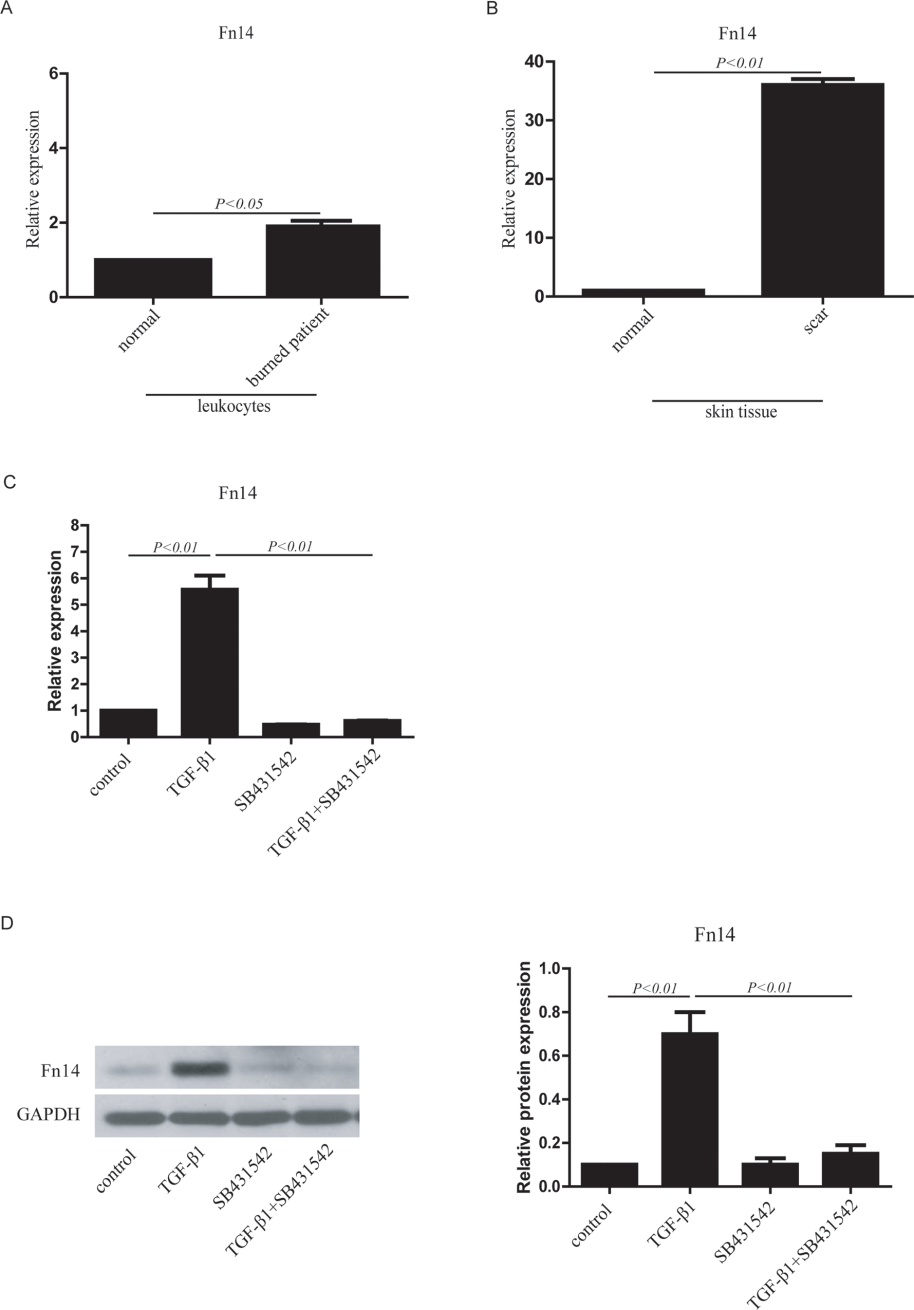
Fn14 expression in human tissue and cultured cells in response to TGF-β signaling. (A) Fn14 expression in leukocytes from healthy donors and burn patients was detected by RT–qPCR. n = 3–5. (B) Fn14 expression in skin tissue from healthy donors (normal) and burn patients (scar) was detected by RT–qPCR. n = 3–5. (C) Fn14 mRNA expression was detected by RT–qPCR in human dermal fibroblasts (HDFs) in response to TGF-β signaling. (D) Fn14 protein expression in human dermal fibroblasts (HDFs) in response to TGF-β signaling was detected by western blotting. Human dermal fibroblasts (HDFs) were treated with TGF-β1, SB431542 or TGF-β1 plus SB431542. The protein expression data in the histogram was calculated using gray scale western blots of HDFs cultured in six-well plates. Total RNA was isolated from human tissues and cultured cells using TRIzol Reagent, and cDNA was synthesized for RT–qPCR. Data from at least three independent experiments are shown. Data are presented as the mean ± SD (standard deviation).

Because previous studies [27, 28] demonstrated a key role of TGF-β in fibrotic responses in animals, the expression of TGF-β1, a member of the TGF-β family, was detected to investigate the role of TGF-β in human fibroblast activation, which can cause skin fibrosis. Patients with thermal burns were selected. Because we focused on fibroblast activation, blood and skin tissues were collected from donors, and the mRNA expression levels of TGF-β1 were examined. We found that TGF-β1 expression in leukocytes from burn patients was higher than that in leukocytes from control patients (S1A Fig). TGF-β1 expression in scar tissue from burn patients was also higher than that in normal tissue (S1B Fig). Because the hypertrophic markers COL1A1 and COL3A1 [29, 30] were detected by RT-qPCR (S1C Fig and S1D Fig), skin tissues from burn patients were classified as hypertrophic scars with fibroblast activation. Recent evidence indicated that fibroblast growth factor-inducible molecule 14 (Fn14) plays an important role in cardiac fibrosis [13–16] and kidney fibrosis [17–19]. However, whether Fn14 plays a role in fibrotic diseases, such as skin fibrosis after burn, is still unclear. In this study, we found that Fn14 expression in leukocytes from burn patients was higher than that in leukocytes from control patients (Fig 1A). Fn14 expression was also higher in scar tissue from burn patients than in normal tissue from control patients (Fig 1B). These results demonstrated a strong association between TGF-β1 and Fn14 expression in leukocytes and scar tissue, and therefore, we hypothesized that there could be an interaction between TGF-β signaling and Fn14.

To identify the function of Fn14 and to ascertain whether TGF-β signaling plays a key role in fibroblast activation through Fn14, we established an in vitro model of fibroblast activation. Dermal fibroblasts from a healthy donor were isolated and treated with TGF-β1, the TGF-β signaling inhibitor SB431542, or TGF-β1 plus SB431542 (S2A Fig). The RT-qPCR analysis indicated that COL1A1 mRNA expression was upregulated in the TGF-β1-treated group compared with the control group (no TGF-β1 treatment), but the effect was reversed by SB431542. SB431542 inhibited COL1A1 mRNA expression in fibroblasts that were co-treated with TGF-β1 (S2B Fig). The mRNA expression of COL3A1 (S2C Fig) was similar to that of COL1A1. Moreover, high protein expression of α-SMA, COL1 and phospho-SMAD2/3 was detected after TGF-β1 treatment, while SB431542 inhibited the expression of these proteins (S2D Fig). Therefore, this was a suitable model of fibroblast activation for studying Fn14 function. In the fibroblast activation model, TGF-β1 upregulated Fn14 mRNA expression, and SB431542 inhibited this response (Fig 1C). Fn14 protein expression was increased by TGF-β1 and decreased by SB431542 (Fig 1D). The data indicated that Fn14 expression was directly regulated by TGF-β signaling in the fibroblast activation model.

### Overexpression of Fn14 promotes the activation of human dermal fibroblasts

**Fig 2:**
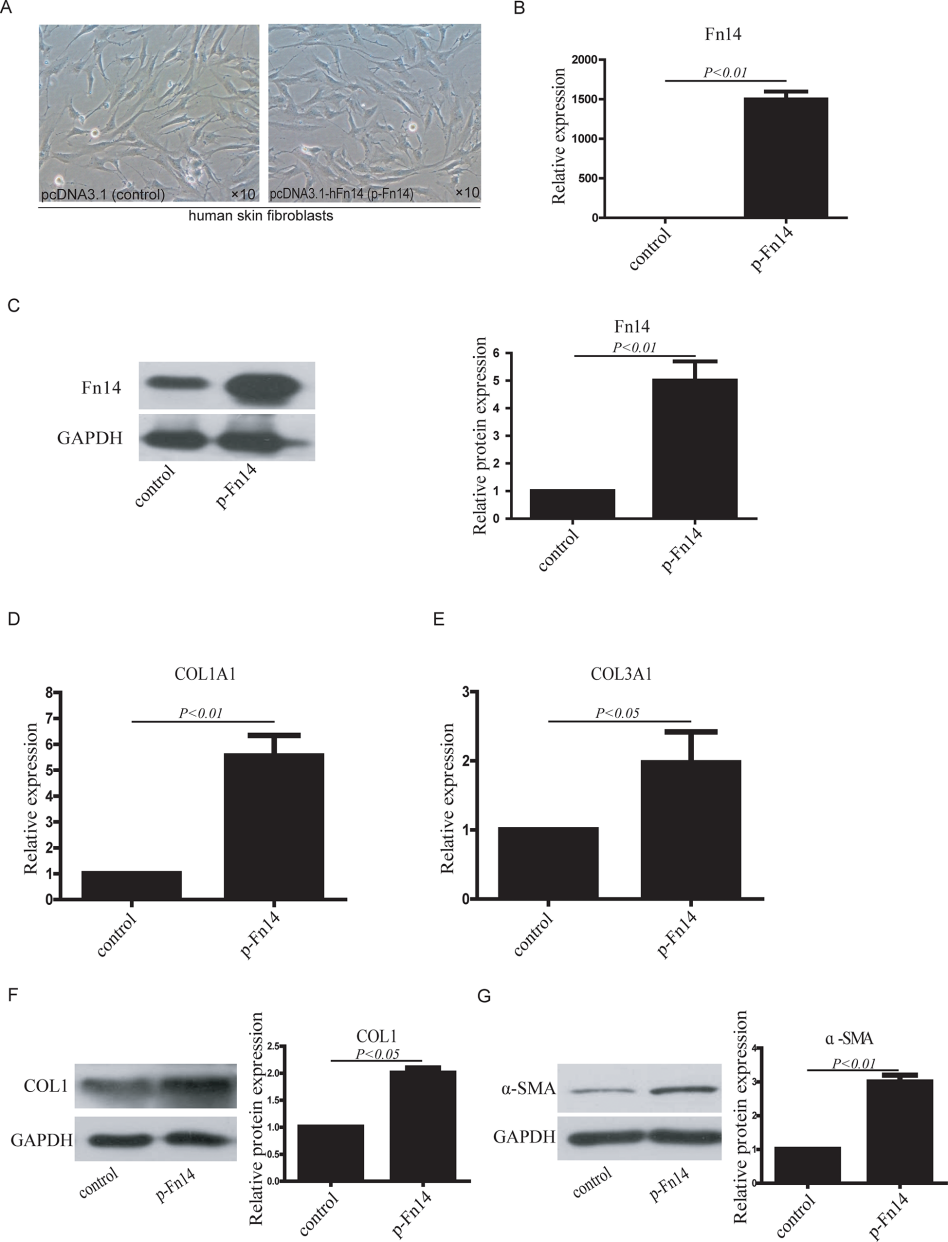
Fn14 overexpression in HDFs promotes fibroblast activation. (A) Cultured HDFs were transfected with pcDNA3.1 (control) or pcDNA3.1-hFn14 (p-Fn14). (B) Fn14 mRNA expression in HDFs was detected by RT–qPCR after Fn14 overexpression. (C) Fn14 protein expression in HDFs was detected by western blotting after Fn14 overexpression. (D) COL1A1 mRNA expression was detected by RT–qPCR. (E) COL3A1 mRNA expression was detected by RT–qPCR. (F) COL1 protein expression was detected by western blotting. (G) αSMA protein expression was detected by western blotting. The protein expression data in the histogram were calculated using gray scale western blots. Data from at least three independent experiments are shown. Data are presented as the mean ± SD.

To further investigate the function of Fn14 in HDFs, Fn14 was overexpressed in HDFs using the pcDNA3.1 vector; empty pcDNA3.1 vector was used as a control (Fig 2A). Fn14 mRNA expression in Fn14-overexpressing HDFs (p-Fn14-HDFs) was significantly higher than that in control HDFs (Fig 2B). Fn14 protein expression in Fn14-overexpressing HDFs was also significantly higher than that in control HDFs (Fig 2C).

To analyze whether Fn14 affects fibroblast activation, RT-qPCR and western blotting were used to detect the expression of collagens and α-SMA. The RT-qPCR analysis showed that COL1A1 mRNA expression was higher in p-Fn14-HDFs than in control HDFs (Fig 2D). COL3A1 mRNA expression also increased significantly in p-Fn14-HDFs compared with control HDFs (Fig 2E). The results also showed that the protein expression of COL1 (Fig 2F) and α-SMA (Fig 2G) was upregulated in p-Fn14-HDFs. Taken together, these results indicate that the overexpression of Fn14 can promote the activation of human dermal fibroblasts.

### Knockdown of Fn14 inhibits the activation of human dermal fibroblasts

**Fig 3:**
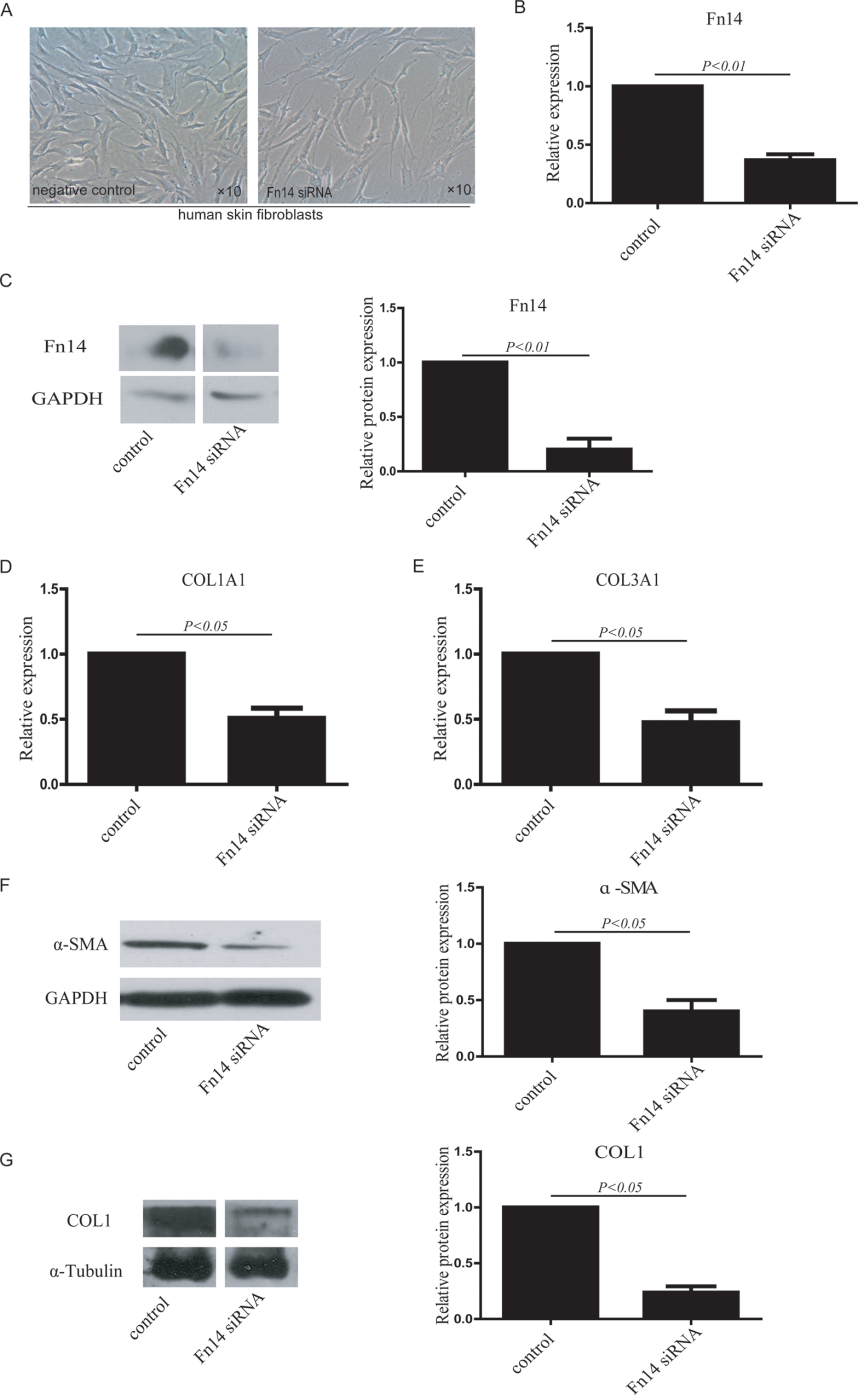
Fn14 knockdown via siRNA in HDFs inhibits fibroblast activation. (A) Fn14 was knocked down in cultured HDFs with Fn14 siRNA, and an siRNA negative sequence was used as a control (negative control). (B) Fn14 mRNA expression was detected in Fn14 siRNA-transfected cells and control cells by RT-qPCR. (C) Fn14 protein expression in Fn14 siRNA-transfected cells and control cells was detected by western blotting. (D) COL1A1 mRNA expression in HDFs transfected with or without Fn14 siRNA was detected by RT-qPCR. (E) COL3A1 mRNA expression in HDFs transfected with or without Fn14 siRNA was detected by RT-qPCR. (F) αSMA protein expression in HDFs transfected with or without Fn14 siRNA was detected by western blotting. (G) COL1 protein expression in HDFs transfected with or without Fn14 siRNA was detected by western blotting. The protein expression data in the histogram were calculated using gray scale western blots. Data from at least three independent experiments are shown. Data are presented as the mean ± SD.

To further analyze the function of Fn14 in HDFs, siRNA was used to knock down Fn14 expression in HDFs (Fig 3A). RT-qPCR demonstrated that Fn14 siRNA significantly inhibited Fn14 mRNA expression (Fig 3B). Western blotting showed that Fn14 siRNA also significantly decreased Fn14 protein expression (Fig 3C). After Fn14 knockdown, COL1A1 expression (Fig 3D) and COL3A1 expression (Fig 3E) were lower compared with control HDFs, as determined by RT-qPCR. α-SMA protein expression in Fn14 siRNA-transfected HDFs was also significantly lower compared to that in control HDFs (Fig 3F). Moreover, COL1 protein expression was significantly downregulated in Fn14 siRNA-transfected HDFs (Fig 3G). These results demonstrate that knockdown of Fn14 decreases the activation of human dermal fibroblasts.

### TGF-β signaling promotes Fn14 transcription through its mediator SMAD4, which can bind to the Fn14 promoter

**Fig 4:**
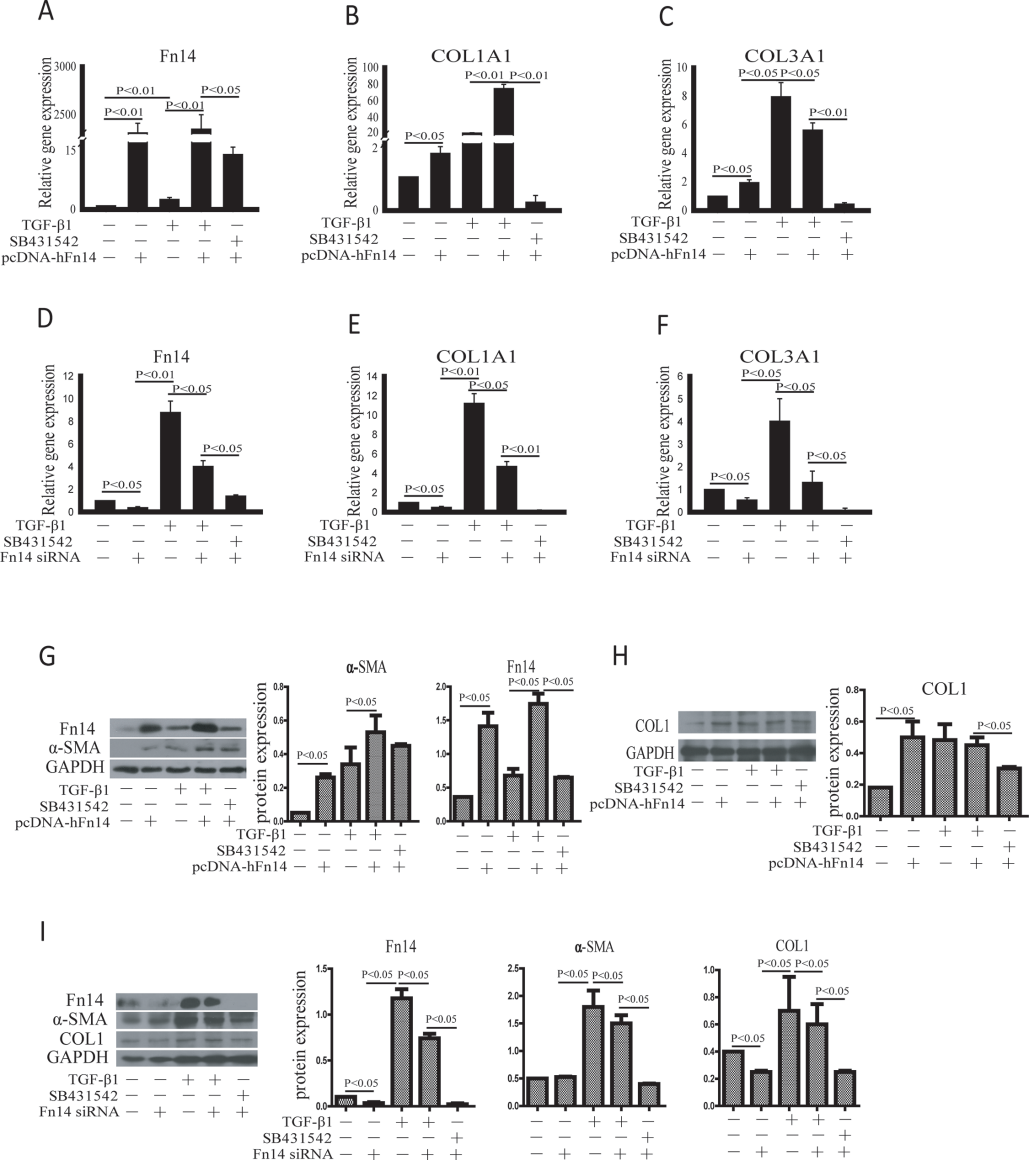
TGF-β signaling promotes fibroblast activation through Fn14 expression. (A)-(F) qPCR was performed to detect the mRNA expression of Fn14 (A and D), COL1A1 (B and E), and COL3A1 (C and F). (G)-(I) Western blotting was performed to detect protein expression. HDFs were cultured in six-well plates. TGF-β1 was used to activate TGF-β signaling, and SB431542 was used to inhibit TGF-β signaling. The pcDNA3.1-Fn14 vector was used to overexpress Fn14. Fn14 knockdown in HDFs was achieved using Fn14 siRNA, and siRNA negative control was used. Data from at least three independent experiments are shown. Data are presented as the mean ± SD.

To investigate how the TGF-β signaling pathway regulates Fn14 expression in fibroblasts, HDFs were exposed to TGF-β1, SB431542, the p-Fn14 vector and Fn14 siRNA. Fn14 mRNA expression was significantly upregulated in cells transfected with p-Fn14 compared to control cells (Fig 4A). Fn14 mRNA expression in TGF-β1-treated cells was also higher compared to that in control cells. Fn14 mRNA expression in cells treated with SB431542 and p-Fn14 was lower than that in cells treated with TGF-β1 plus p-Fn14 (Fig 4A). Both TGF-β1 and p-Fn14 promoted COL1A1 (Fig 4B) and COL3A1 (Fig 4C) mRNA expression. Fn14 mRNA expression decreased significantly after Fn14 siRNA transfection (Fig 4D). The mRNA expression of both COL1A1 (Fig 4E) and COL3A1 (Fig 4F) was significantly downregulated in Fn14 siRNA-transfected cells compared to control cells. Although the TGF-β signaling pathway is activated by TGF-β1, Fn14 siRNA inhibited Col 1α1 (Fig 4E) and Col 3α1 (Fig 4F) mRNA expression compared to TGF-β1 treatment. Then, protein expression was evaluated. Fn14 protein expression was promoted by TGF-β and decreased by SB431542 (Fig 4G). The protein expression of α-SMA (Fig 4G) and COL1 (Fig 4H) was upregulated by TGF-β and downregulated by SB431542. Although the TGF-β signaling pathway is activated by TGF-β1, Fn14 siRNA inhibited α-SMA and COL1 protein expression (Fig 4I) compared to TGF-β1 treatment. Thus, these data indicated that Fn14 indeed plays an important role downstream of the TGF-β signaling pathway, which promotes fibroblast activation. However, how Fn14 expression is regulated by TGF-β signaling is still unknown.

**Fig 5:**
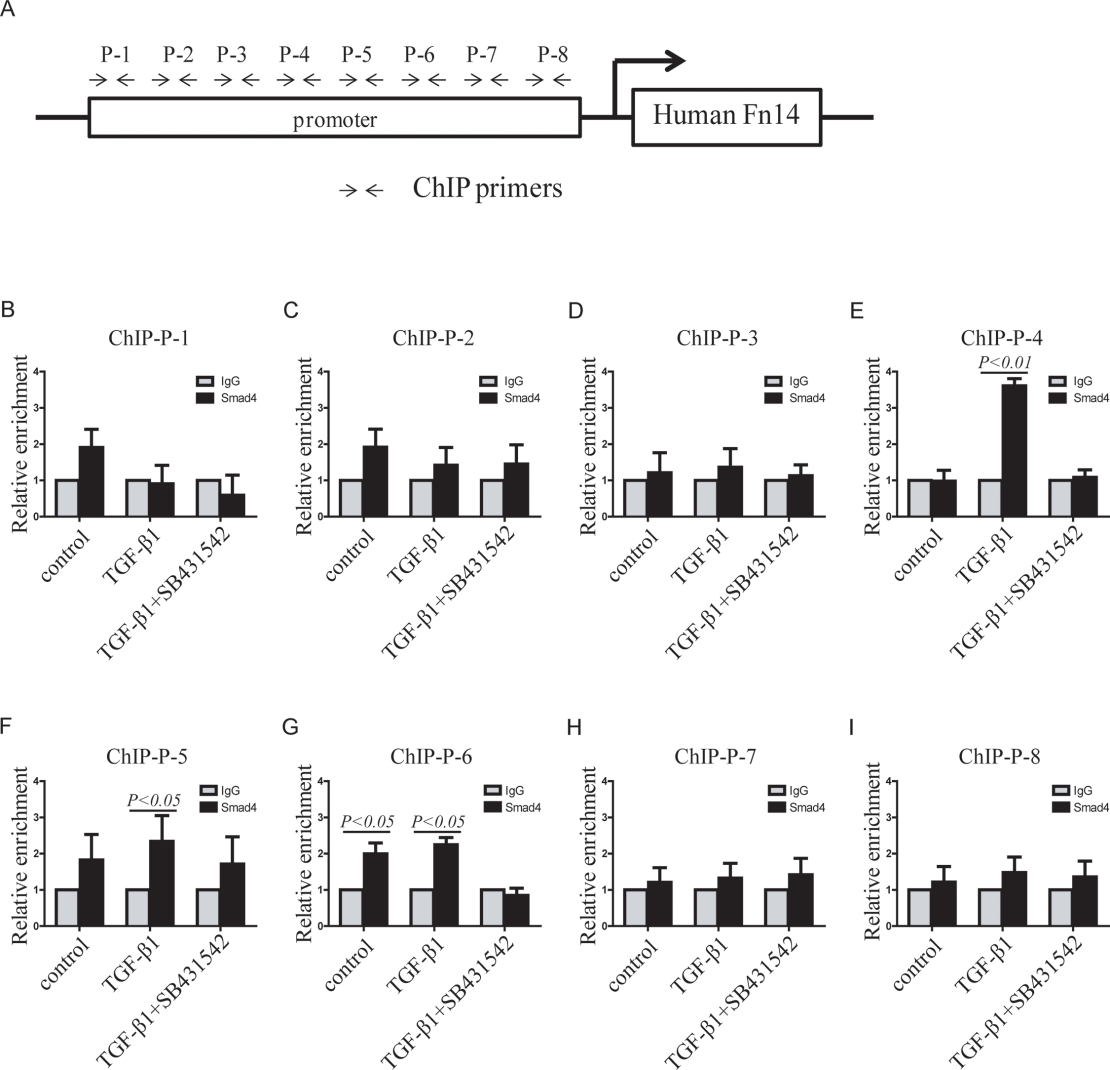
Detection of SMAD4 binding to the human Fn14 promoter in HDFs. (A) The human Fn14 gene promoter was analyzed, and primers for chromatin immunoprecipitation (ChIP) were designed according to the sequences of potential binding sites. (B)-(I) ChIP-qPCR was performed with primer sets P-1 (B), P-2 (C), P-3 (D), P-4 (E), P-5 (F), P-6 (G), P-7 (H) and P-8 (I). Data from at least three independent experiments are shown. Data are presented as the mean ± SD.

It was reported that SMAD4 regulates specific gene transcription through binding to gene promoters [31–33]. The data in Fig 1C and 1D showed that Fn14 was significantly upregulated by TGF-β signaling in HDFs. So, we analyzed the subcellular localization of SMAD4, which is an important co-mediator in the TGF-β signaling pathway. Immunocytochemistry results showed that SMAD4 protein was localized to both the cytoplasm and nucleus of HDFs. SMAD4 translocated into the nucleus of HDFs after TGF-β1 treatment. SMAD4 protein remained in both the cytoplasm and nucleus of HDFs treated with TGF-β1 plus SB431542 (S3 Fig). Therefore, we hypothesized that SMAD4 plays an important role in Fn14 expression. Then, we analyzed the promoter sequences of the human Fn14 gene and discovered “CAGA” boxes (S1 Text). Because the “CAGA” box has been reported as a basic SMAD4 binding box [34], we postulated that SMAD4 binds to the Fn14 promoter and promote its transcription. To verify this, chromatin immunoprecipitation assays (ChIP assays) and qPCR were used to detect SMAD4 binding. We designed 8 primers pairs against the “CAGA” boxes in the human Fn14 promoter. These 8 ChIP primers pairs were named P-1, P-2, P-3, P-4, P-5, P-6, P-7, and P-8 (Fig 5A). The qPCR results demonstrated that only the P-4, P-5 and P-6 primers had signals that responded to TGF-β1 or TGF-β1 plus SB431542 (Fig 5B–5I). As evidenced by the P-4 qPCR result, SMAD4 did not bind to the promoter at this site in control HDFs (Fig 5E); however, TGF-β1 significantly increased SMAD4 binding to the promoter, whereas SB431542 reversed the effect of TGF-β1 and inhibited SMAD4 binding to the Fn14 promoter (Fig 5E). These data suggest that this site in the Fn14 promoter is an inducible binding site of SMAD4 that is responsive to the TGF-β signaling pathway. As evidenced by the P-5 qPCR result, SMAD4 remained bound to the promoter at this site with or without TGF-β1 or SB431542 (Fig 5F). These data indicated that this site in the Fn14 promoter may not be an inducible binding site but rather a constitutive or basic SMAD4 binding site that is unresponsive to the TGF-β signaling pathway. We found that SMAD4 bound to the promoter at the P-6 site (Fig 5G); however, TGF-β1 did not increase SMAD binding to this site, and SB431542 completely blocked SMAD4 binding (Fig 5G). These findings suggested that this site in the Fn14 promoter is an inducible SMAD4 binding site that is responsive to the TGF-β signaling pathway. These results showed that SMAD4, an important TGF-β signaling mediator, can bind to the Fn14 promoter.

Taken together, Fn14 is an important factor that is downstream of the TGF-β signaling pathway. Furthermore, TGF-β signaling promotes fibroblast activation through Fn14.

## Discussion

In this study, we elucidated the role of Fn14 in fibroblast activation and ECM synthesis in human dermal fibroblasts. We established an in vitro model of HDF activation induced by TGF-β to investigate how the TGF-β signaling pathway regulates ECM synthesis and fibroblast activation, which are enhanced in skin fibrosis. We found that Fn14, a downstream factor of the TGF-β signaling pathway, promoted ECM synthesis by activation of HDFs.

Collagen accumulation or ECM synthesis is the hallmark of skin fibrosis. Several polypeptide growth factors regulate tissue repair and fibrosis [8, 35]. TGF-β is a well-known inducer of collagen synthesis. TGF-β signaling is involved in fibroblast activation during chronic fibrosis; for example, there is evidence that it plays a key role in SSc fibrosis [24, 36, 37]. TGF-β expression is involved in burns, which are the leading cause of hypertrophic scarring [38]. TGF-β signaling increases human fibroblast activation and ECM production [39–41]. However, further details regarding the regulation of collagen or ECM synthesis by TGF-β signaling remain unclear. Here, we established an in vitro skin fibrosis model using human dermal fibroblasts treated with TGF-β1. In most cell types, TGF-β regulates collagen via the canonical SMAD pathway by binding to and activating specific type I and type II serine/threonine kinase receptors. This results in the phosphorylation and activation of SMAD2/3, followed by its nuclear translocation [42]. Here, we also found that SMAD4, another important mediator in the TGF-β signaling pathway, played an important part in fibroblast activation. Most importantly, we discovered that Fn14 regulated fibroblast activation. Although Fn14, which was identified as the tumor necrosis factor-like weak inducer of apoptosis (TWEAK) receptor, was reported to play an important role in other diseases, such as cardiac hypertrophy [43], cardiac failure [13], skeletal muscle atrophy and metabolic dysfunction [21], there are no reports on Fn14 function in human fibroblast activation. In this study, we found that Fn14 was a novel inducer of fibroblast activation.

In the human Fn14 promoter, certain SMAD4 binding sites were predicted. ChIP was used to confirm that SMAD4 indeed bound to the Fn14 promoter and activated its transcription, which has not been reported previously. High levels of Fn14 mRNA have been detected in heart, kidney, lung and placenta [12, 44]. Our group also discovered that Fn14 promotes the differentiation of human mesenchymal stem cells into heart valvular interstitial cells by phenotypic characterization [45]. Here, we used gain-of-function and loss-off-function methods to discover that Fn14 played an important role in ECM synthesis and human dermal fibroblast activation in response to the TGF-β signaling pathway. Fn14 is also a receptor for TWEAK, which is a multifunctional cytokine that promotes cell death, cell proliferation, inflammation, and angiogenesis [46, 47]. The TWEAK/Fn14 signaling pathway is also involved in many biological functions and diseases [48–50]. Other potential interactions between the canonical TGF-β signaling pathway and the canonical TWEAK/Fn14 signaling pathway as well as the mechanism by which Fn14 regulates fibroblast activation must be studied in the future.

**Fig 6:**
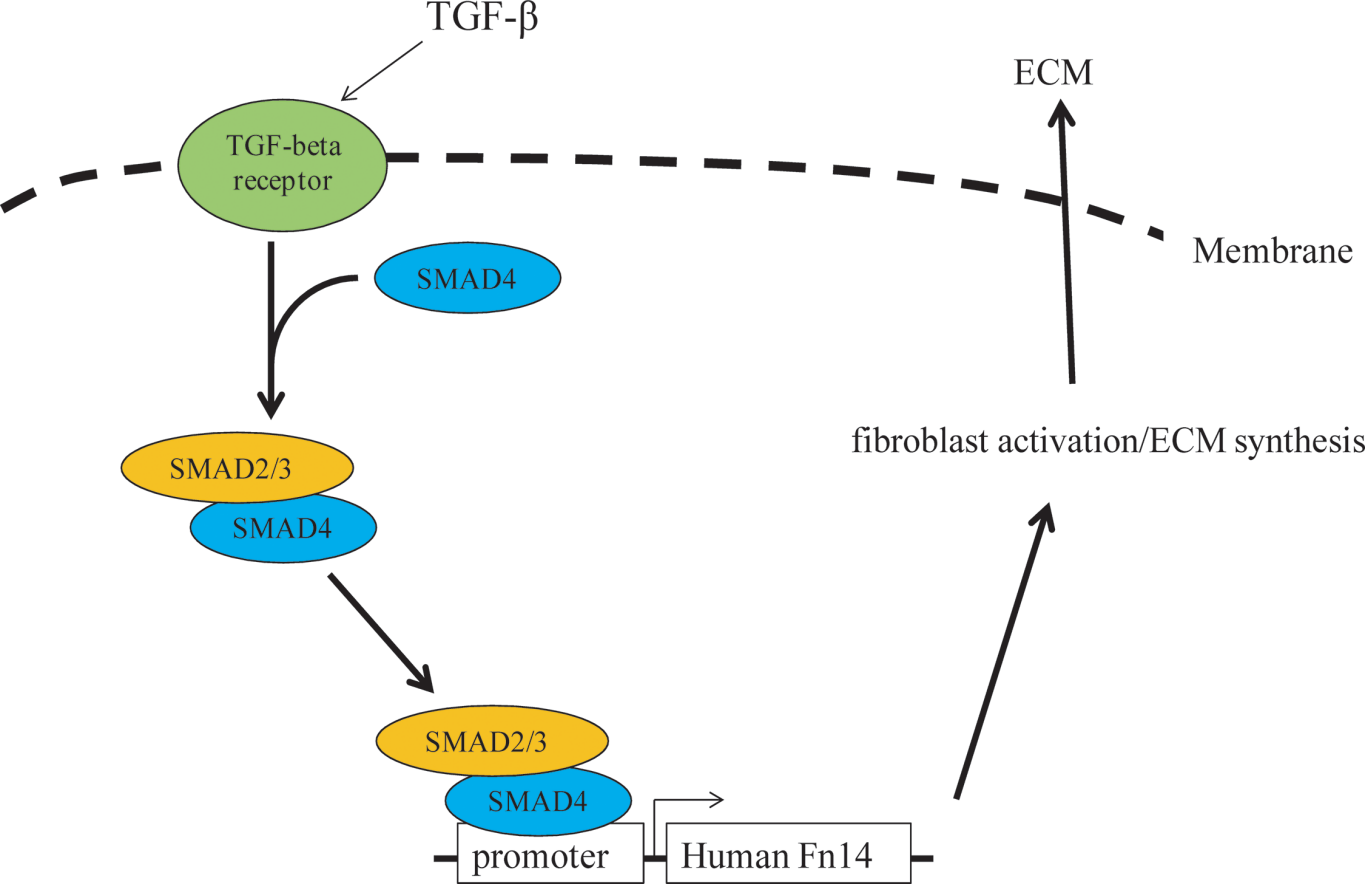
Fn14, a downstream target of the TGF-β signaling pathway, regulates dermal fibroblast activation. The TGF-β signaling pathway increases Fn14 expression through SMAD4 binding to the Fn14 promoter in human dermal fibroblasts. Then, upregulated Fn14 expression promotes ECM synthesis and fibroblast activation.

Taken together, our study demonstrated that Fn14 is an important factor that is downstream of the TGF-β signaling pathway. SMAD4-induced Fn14 expression can promote ECM synthesis and fibroblast activation. In general, the TGF-β signaling pathway activates the SMAD4 complex through TGF-β receptors. Then, the SMAD4 complex translocates into the nucleus, binds to the Fn14 promoter and activates Fn14 transcription, which further promotes ECM synthesis by activation of human dermal fibroblasts (Fig 6). Our study also indicated that Fn14 is a potential therapeutic target and provided a promising approach for preventing excessive collagen or ECM accumulation in skin fibrosis.

## Supporting Information

S1 Fig mRNA expression of TGF-β1, COL1A1 and COL3A1 in human tissues. (A)-(B) qPCR was used to detect TGF-β1 expression in leukocytes from blood (A) and skin tissue (B). (C)-(D) qPCR was used to detect collagen expression: COL1A1 (C) and COL3A1 (D). Blood and skin tissues were collected from healthy donors (n = 3) and burn patients (n = 5) in the hospital. Total RNA was purified from peripheral blood mononuclear cells (PBMCs) in blood and skin tissues. qPCR was performed with cDNA after reverse transcription from total RNA. Data from at least three independent experiments are shown. Data are presented as the mean ± SD (standard deviation).
(TIF)

S2 Fig TGF-β signaling promotes the activation of human dermal fibroblasts (HDFs). (A) HDFs were treated with TGF-β1 or the TGF-β signaling inhibitor SB431542. (B)-(C) The mRNA expression of COL1A1 (B) and COL3A1 (C) was detected by RT-qPCR in HDFs in response to TGF-β signaling. (D) The protein expression of factors downstream of TGF-β signaling and of fibroblast activation makers was detected by western blotting. Data from at least three independent experiments are shown. Data are presented as the mean ± SD.
(TIF)

S3 Fig The effect of the TGF-β signaling pathway on SMAD4 protein localization in HDFs. HDFs were cultured in six-well plates. Cells treated with or without TGF-β and SB431542 were subjected to immunohistochemistry with the SMAD4 antibody (green) and analyzed by confocal microscope. Nuclei (blue) were stained with Hoechst 33342. Scale bar, 50 μm.
(TIF)

S1 Text Supplementary materials and methods. (DOCX)

## Abbreviations

TGF-β: transforming growth factor-β
Fn14: fibroblast growth factor-inducible molecule 14
SMAD4: SMAD family member 4
HDFs: human dermal fibroblasts
ECM: extracellular matrix
