# Supplementary figures and images for: Fn14, a Downstream Target of the TGF-β Signaling Pathway, Regulates Fibroblast Activation
Source: PLoS One. 2015 Dec 1;10(12):e0143802. doi: 10.1371/journal.pone.0143802 (PMC4666639; doi:10.1371/journal.pone.0143802)

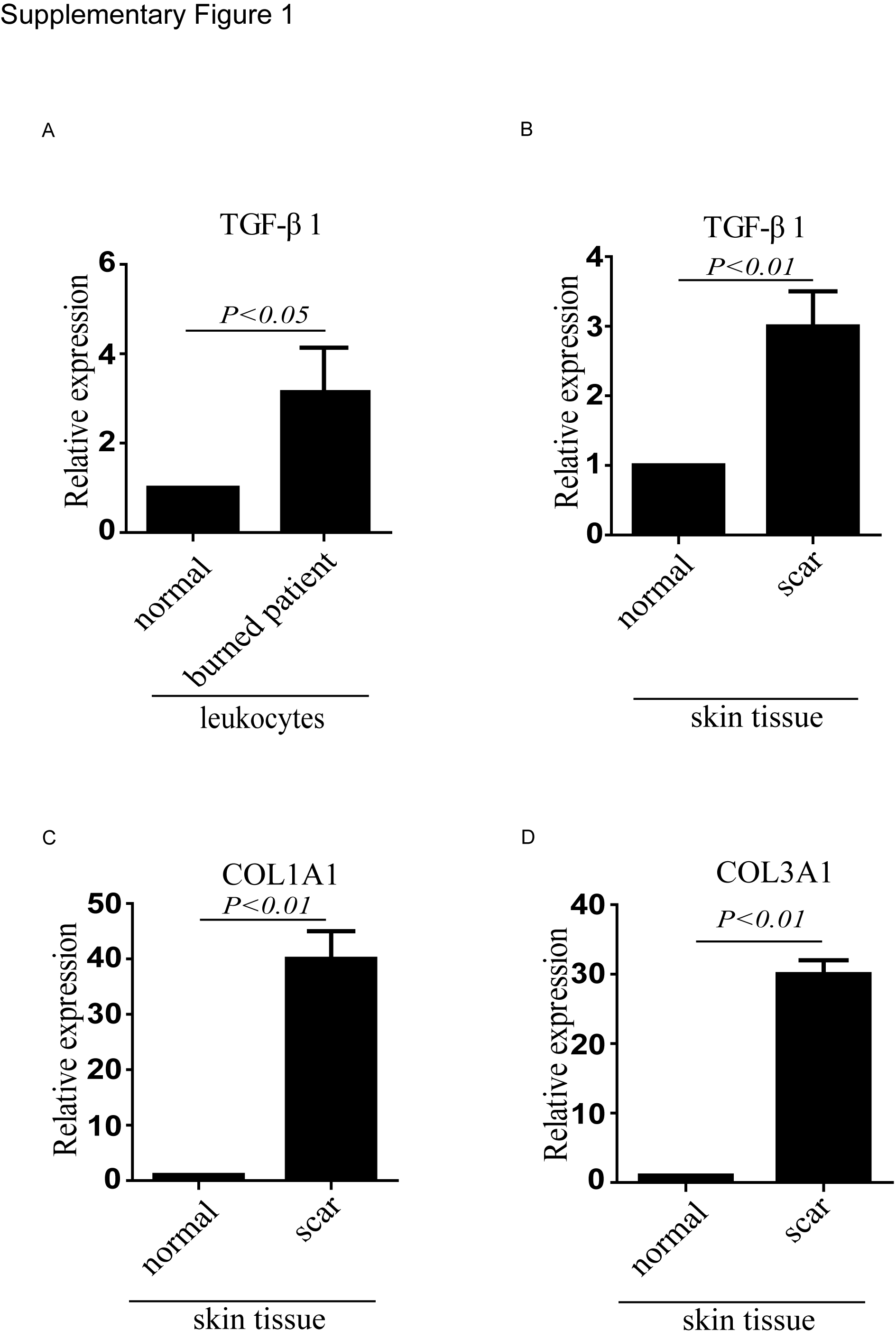

Supplement: S1 Fig — (A)-(B) qPCR was used to detect TGF-β1 expression in leukocytes from blood (A) and skin tissue (B). (C)-(D) qPCR was used to detect collagen expression: COL1A1 (C) and COL3A1 (D). Blood and skin tissues were collected from healthy donors (n = 3) and burn patients (n = 5) in the hospital. Total RNA was purified from peripheral blood mononuclear cells (PBMCs) in blood and skin tissues. qPCR was performed with cDNA after reverse transcription from total RNA. Data from at least three independent experiments are shown. Data are presented as the mean ± SD (standard deviation). (TIF) [file pone.0143802.s001.tif]

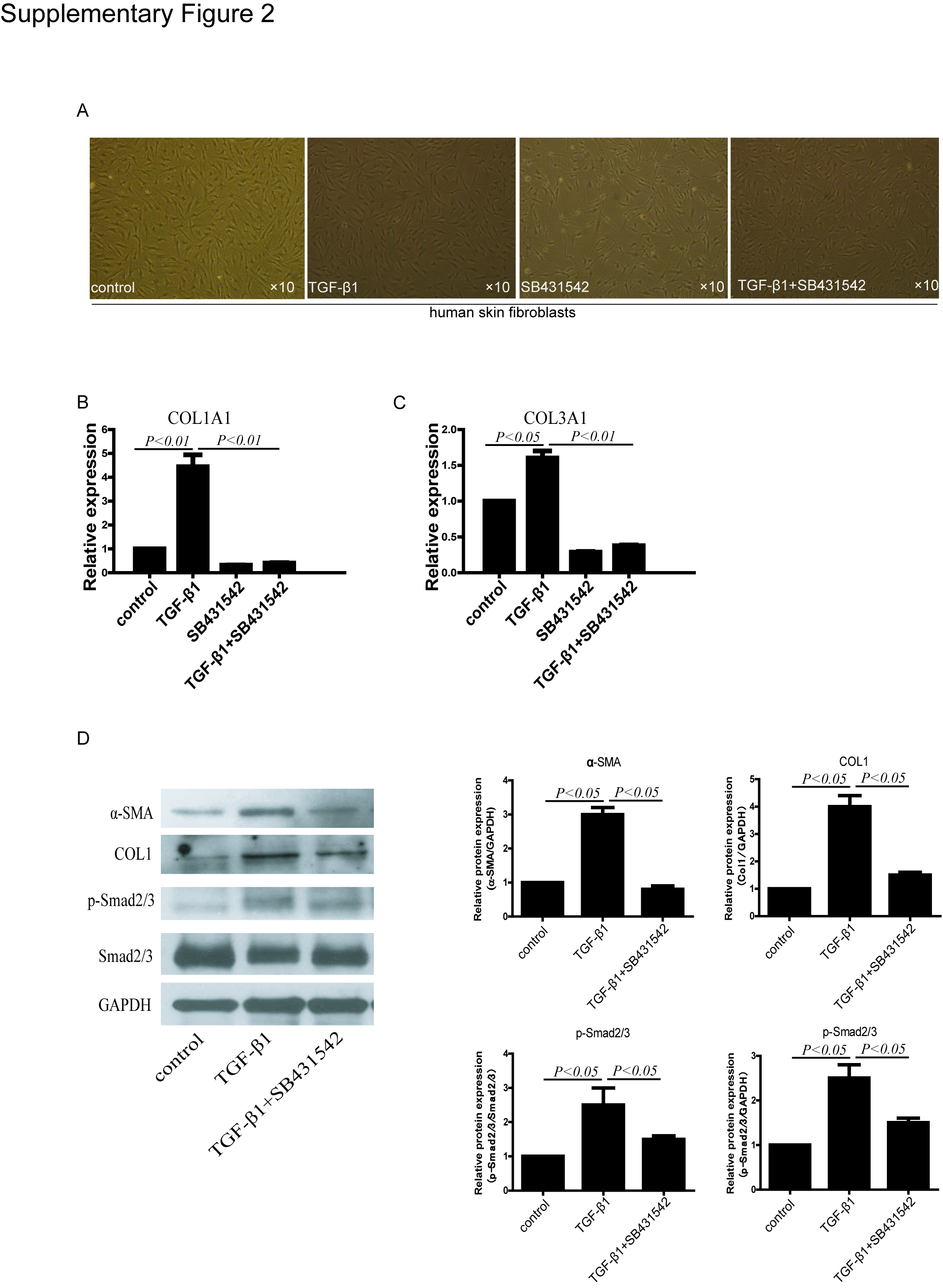

Supplement: S2 Fig — (A) HDFs were treated with TGF-β1 or the TGF-β signaling inhibitor SB431542. (B)-(C) The mRNA expression of COL1A1 (B) and COL3A1 (C) was detected by RT-qPCR in HDFs in response to TGF-β signaling. (D) The protein expression of factors downstream of TGF-β signaling and of fibroblast activation makers was detected by western blotting. Data from at least three independent experiments are shown. Data are presented as the mean ± SD. (TIF) [file pone.0143802.s002.tif]

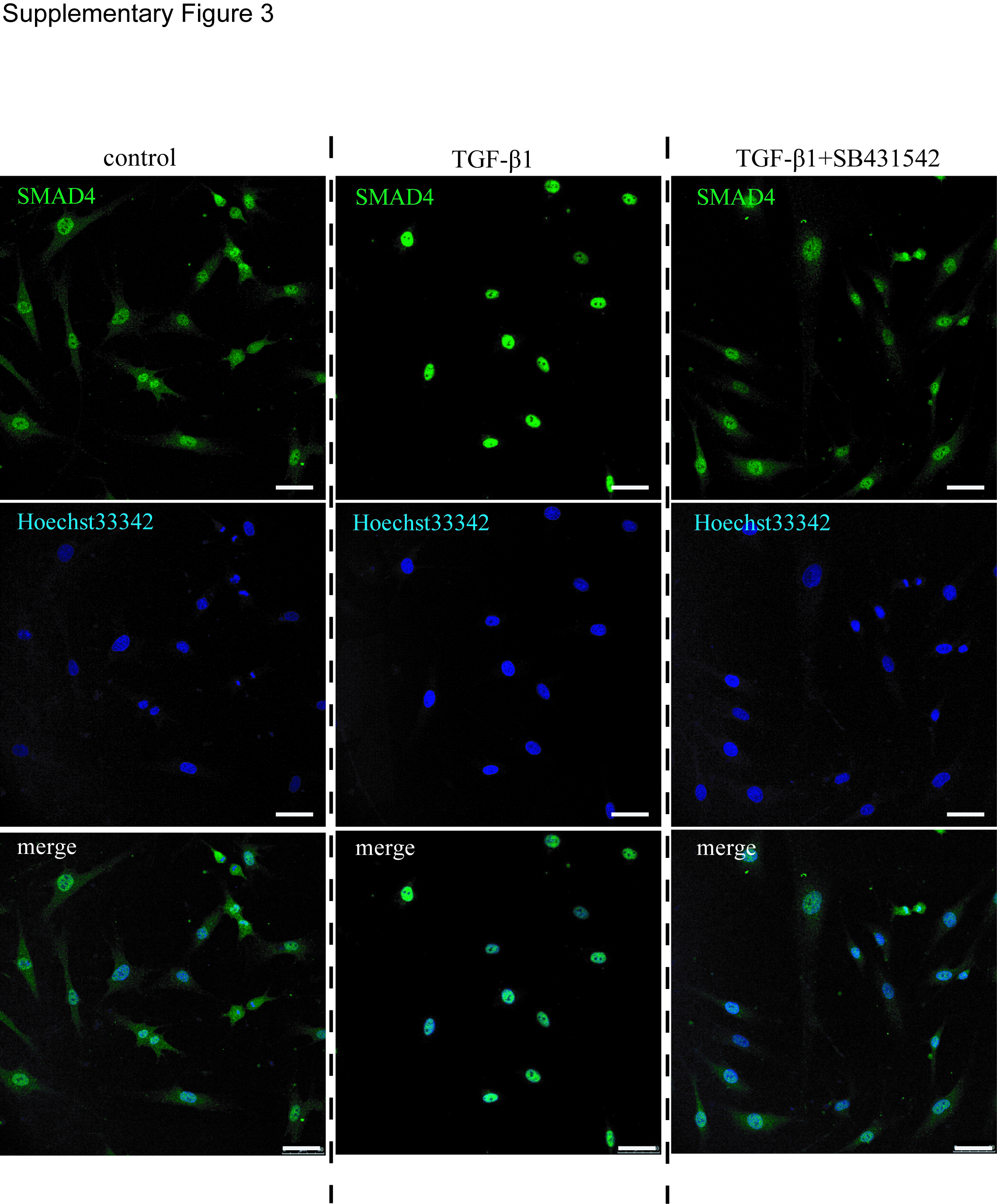

Supplement: S3 Fig — HDFs were cultured in six-well plates. Cells treated with or without TGF-β and SB431542 were subjected to immunohistochemistry with the SMAD4 antibody (green) and analyzed by confocal microscope. Nuclei (blue) were stained with Hoechst 33342. Scale bar, 50 μm. (TIF) [file pone.0143802.s003.tif]
