# Supplementary material for: Fn14, a Downstream Target of the TGF-β Signaling Pathway, Regulates Fibroblast Activation
Source: PLoS One. 2015 Dec 1;10(12):e0143802. doi: 10.1371/journal.pone.0143802 (PMC4666639; doi:10.1371/journal.pone.0143802)
Supplement: S1 Text — (DOCX) [file pone.0143802.s004.docx]

**Fn14, a new downstream target of TGF-β signaling pathway, regulates fibroblast activation**

**Supplementary Materials and Methods**

**Immunocytochemistry**

The intracellular localization of endogenous SMAD4 was examined by immunocytochemistry and fluorescence confocal microscopy (Leica, Germany). Cultures were fixed with paraformaldehyde and then stained with primary antibody to SMAD4, incubated with secondary antibody. Nuclei were identified with Hoechst33342. Subcellular distribution of fluorescence was evaluated by laser scanning confocal microscopy. Each experiment was repeated at least three times with consistent results. The information of all antibodies used in this study is given in Supplementary materials.

**Specific RT-qPCR primers for Col1α1, Col3α1, β-actin, TGF-β1 and Fn14 were as follows:**

COL1A1 forward: AAGGTGTTGTGCGATGACG,

COL1A1 reverse: TGGTCGGTGGGTGACTCTG;

COL3A1 forward: GAGCTGGCTACTTCTCGC,

COL3A1 reverse: TCTATCCGCATAGGACTGAC;

β-actin forward: gccaacacagtgctgtctg,

β-actin reverse: tactcctgcttgctgatcca;

Fn14 forward: CGACCGCACAGCGACTTC,

Fn14 reverse: GGCATCGTCTCCAGACCAAA

TGF-β1 forward: ACCCACAACGAAATCTATGACA,

TGF-β1 reverse: GCTGAGGTATCGCCAGGAAT,

**Specific qPCR primers in ChIP assay were as follows:**

P-1 forward: cagaatctcaaagcatcccc,

P-1 reverse: tctgcctcctgctatttttc;

P-2 forward: ttctggatggctctagcgcca,

P-2 reverse: gcccgctactcaacatctgc

P-3 forward: cagaggtgagcaccaggcgggc,

P-3 reverse: acgtgccctctgtgtcatcgc;

P-4 forward: gatctgcattccggcagaggc,

P-4 reverse: atgtggcctgaggtctgggcc.

P-5 forward: ggaggaggggcagagaccctg,

P-5 reverse: gtctcaagctctgttctaccc,

P-6 forward: ggccagataatattatttttatg,

P-6 reverse: ctctggaggctgaggcaggtg,

P-7 forward: atacaaaaattagccggac,

P-7 reverse: gctcagcctggagtgcagtgg,

P-8 forward: accacgcccggctaatttttg,

P-8 reverse: ggctcacgcctttaatccc,

**The sequences of all siRNAs were as follows:**

Negative control (siRNA control): aacgtacgcggaatacttcgatt

Fn14 siRNA: gagggagaatttattaataaa.

**The information of all antibodies was as follows:**

Rabbit anti-SMAD4 (Cell Signaling Technology, USA)

Mouse anti-α-SMA (DAKO, Denmark)

Rabbit anti-COL1 (Abcam, British)

Rabbit anti-p-Smad2/3 (Cell Signaling Technology, USA)

Rabbit anti-Fn14 (Cell Signaling Technology, USA)

HRP conjugated anti-GAPDH (KangCheng, China)

Alex Flour FITC anti-rabbit (Life technologies, USA)

Goat anti-mouse (Earthox, USA)

Goat anti-rabbit (Earthox, USA)

Rabbit IgG (Cell Signaling Technology, USA)

Alexa Fluor@488 donkey anti-mouse IgG (H+L) (molecular probes, USA)

**Fn14 promoter sequences information:**

Promoter sequences of human Fn14 (from +1 to -5313 bp, total 5314bp):

“caga” shown in sequences below is predictive basic binding site of SMAD4. ChIP-qPCR primer pairs P1, P2, P3, P4, P5, P6, P7 and P8 are designed according to the sequences marked with lines below, respectively.

ccagcccccagcagcagcaggaactcttggggacagtctgtcttgttgcaaagccagcacagcaagcagcctccgcatta

gttccatagcttgactggcttctaagatgggcatgtcaagatccagaatctcaaagcatcccctctttggctccatcatc

caagggtgagaaacagcagagcctaagtgagagtctgagtcaacaccttggctcagttttcaaatgaattttaaatatcc

tcagtcaaaagaaaaatagcaggaggcagaaacaaaaggtacgaacccatcccaaagctgttgggcactgccacttctgg

atggctctagcgccagcggagcccccattcccctccacgtcagacgtaattcttggtagggtactcagagggcccccgag

agatggcaggggcagatgttgagtagcgggccatgtaatggctggggccctgggacccccccgaggggcaagtgcagcac

agcaaccccccacccagcaacaaaaggcctgaggccgcccagcccaagtagagggaggcccccagctcccgcttttgggc

ctcagccaccagggggttatagaagtcccggatgatggcatgcgccgtccagcacacggggattagcgtcaggacccctg

agatgacaaagacaatcccagaggtgagcaccaggcgggccttggaatccttctcctccacacaggtggtacacttggcc

ccagcaaggtagaccagcaagccgaacagggccacaaggagggcgatgacacagagggcacgtgcagcctgcaggtcctg

tggcagcgccagcagtgagtcgtacaccttgcactgcatctggccggtgctctgcaccacgcaggacatccacaggccct

cccacaccacctgggccaccacgatgctgttgccgatgaaagcggtcaccttccacatgggcagggcacaggagaccagg

ccattcacccagcccagcagtgtcaggacgactcccaggatctgcattccggcagaggccatggcgaggttgaaggagct

gcactgtgtttgggacagaagcacaacaaggtgaggcctggcaggcccagacctcaggccacatgtggacaggactctag

gggcatgcaccctggaaagtggtcatcacatcctggaactcagtcaagacatgctccctgagcctcacttctcgtgggca

aaatggacacttaatgctttagcatttattattctttctagttatgtgacaggcactggctgatgacagtgactgtgcaa

gcagccctgccctggctatcctgaatgcaaatgaaagataacaggatgaaacctatacaatgacagtccaaggccagagc

cagtgatcaggctgcaaggcggcaaaaagcaggatgacaggacggtgcgatttccaagcagtttccgtgtagaatggttc

aattctagccctgagtttaaggctcaaaacttgtccatttgttttgtttgtttgtttgagacgaggagtctcgctctgtg

gcccaggctagagtgcagtggtgccatctccactcactgcaacctctgcctcccaggttcaagcgattctcctgcctcag

cctcccaagtagctgggattacaggtgcacaccaccacgcccggctaattttttttttttttttttaagacggagtctcg

ctctgtcgcccagcctggagtgcagtggcgggatctcggctcactgcaagttctgcctccccggttcacgccattctcct

gcctcagcctcccgagtagctgggactacaggcgctcaccaccacgcccggctaatttttttgtatttttagtagagacg

gggtttcactgtgttagccaggatggtctcgatctcctgacttcgtgatcctcccgcctcagcctcccagagtgctggga

ttacaggcatgagccaccacacctggtcttaatttttgtatttttagtagaaacagggtttcatcaagttggccaggcgg

gtctcgaactcctgacctcaggtgatctgcccgcctcaagcctcccaaagtgctgggattacaggtgtgagccaccgtgc

ccaaccaaaacttgttaatttcaacactaagattggagatcctaagcttcctcccaccacccaatgtccctcacaacttg

gttccacaccccaggctggcagacaccaggaccaggagagatgaagaaaagcatcattaatcccccatctaaaagcagtt

cctcccccaaagccagtggataactccctggccccacccttctgacctgccctaggcgccggagagcatagcctcaaggc

tgagggccttttcctatgaattctagacgaaaatgccgtgctagactcccctcaacaccagttctcccaccgccacctgg

cctgcacctgtgtcccctcagtgttggaggtcgtggagggaatcacttctgactagggccgggctgtggcctcccactgc

ccagagtactttcattgtacggcttggacctaaacttttcccggccggcagcggtcggagggcaggctctactcattagc

cgggggccccaggcctgggcggagcagaccaccttggggagactggctggggcacggtgccccagcgctcaggtccggcc

cttaggcctctggccgagttagggtcggggccgcgctggtataggcgcccaggcctctcccctctgagtgtgcccctgcc

cctttctttttcatgcactgcatgaaaaagactctgtccgagggcgccccggcccacccgaccaccccagcctgggagcc

gcgcgccctggaggggttctatagctgcagtggccgcaaggtccgctcccccaggtgagggggaggggggaggggcgccc

cgccccgagaggcaaggcaggtgggggaggggcgccaggggtttcggagtctttgtccctggggaggggggaggggcgtt

taacccttgggagccccgaaggaccctatcacctcggaggcttgggcgcggaccggccgaccccctcagggactcgccca

tccaggtggccgcagcccgagttcgcacgcacccctacccacgccgccaccaccctgcacccggccccaagaggcgtctc

tccaacacgcacactagccccggactcacctgcgaaggagataagggaaattcctaggccgagtgtcgggacaggggcgg

aggcgggggtcttaaagaagcggtgacgtcactggaccaccgccccggaggaggggcagagaccctgtccctgacggcag

cgggcgtgcgaatcccaggcttgtcagaggggggcgctggaggctggggtcgctgggcccgcctcgggctggcagacgtc

cagactcacccagggtgagggcgccaggcttagggtagaacagagcttgagactccgcttcaccacttaagcagcgcggt

gacatggggaactcacctaaccttcccgagccttcgtttcctactctgcaaaatggagataacaagtctgcattttatta

tgtaactacgtcctaaggtacaaattaaagggcatactattggtattattttagagaaatattttcactgtgtcacccag

gctggagtgcagtggtgcgatcacggctcactgcagtctcgaccttctgggctcaagcgatcctcccgcctcagcctcct

gagtagctgggactacaggcagcacacgccaccgtgcccagctaatttatgttttgttttgtatagacagggtcttgtta

tattgcccaggcgtctcgaactcccggactcaagcgatccgcccgccttggcctcgcactgcgctgggattaccggctta

agccaccgcgcccggccagataatattatttttatgttgctggttttttgttgttgttgttgtttgtttttgagatggag

tctcgctctctcgccaaggctggagtgtggtggcgcgatcttggctcactgcaagctctgccttccgggttcacgccatt

cacctgcctcagcctccagagtagctgggactactacaggtgcccgccaccacccccgactaatttttttgtatttttag

tagagacggggtttcaccgtgttagccaggatggcctcgatttcctgacctcgtgatctgcccgcctcggcctcccaatg

tgctgggattacaggcgtgagccaccgcgcccggcctgttttgttttttgagatggaggccctctctgtcgcccagtctg

gagtgcaatggcgctatctcgactgactgcaacctccgtctcccaggttccagaaattctcctgcctcagtctcccgagt

agctgagattacaggcgcccactaccacgcccggctaacttctttcttttttttatttttagtaaagacgggggttcacc

aagttggccaggctggtctcgagctcctgacctcaggtttaattgttataccccccgggcccttaacaaagctgtagtgg

ccaggctaacctgggcaacatagtaagaccctggtctctacaaaaaatacaaaaattagccggacgtggtggggcagggc

ctgtggtcccagctacttgggaggctgaggtgggaggctcacctgagcccagagagccgtgatcgtaccactgcactcca

ggctgagcaagagtgagacgcaatctcaaggggaggtggggggaaggaagctgcagtgatctctgcatggattggtcgtg

tgtctgatttctttttattgtttgtttgtttgtttatttatttatttatttatttatttatttatttgagacggactctc

actctgtcgcccaggctggagtgtagtggcgcgatctcggctcactgcaacttctgcctcccgggttcaagagattctcc

tgcctcagcctcctgagtagctcggattgcaggcgcgcgccaccacgcccggctaatttttgtatttcgagtagagacgg

cgtttcaccacgttggtcaggctgctctcgaactcctgacctcgtaatccgcctgcctcagactcccaaactgatgggat

taaaggcgtgagccaccgcgtccggccgttcgtgtctgatttctatatgtgctgccgaagcgagcactcgtgtctaattt

ttgatttccccacaccacgcacgctggactagatcaccggctgggcaaggaagggggtctgcgtccctgcggggtcctgg

cagctcccgcgccaggactttgttgaatgaatgactgaactgagcgggcgccgcggggcggggcgtcccgaagcggacct

caaggcgggcggaggcgagagctccgccccggaa
